# Risk factors associated with severe recurrent respiratory papillomatosis

**DOI:** 10.4102/sajid.v34i1.69

**Published:** 2019-11-20

**Authors:** Muddaseer Khan, Tesuven K. Naidu

**Affiliations:** 1 Department of Otorhinolaryngology (ENT), Nelson R Mandela School of Medicine, University of KwaZulu- Natal, Durban, South Africa; 2 Department of Otorhinolaryngology (ENT), General Justice Gizenga Mpanza (GJGM) Regional Hospital, Durban, South Africa; 3 Department of Otorhinolaryngology, Bay of Plenty District Health Board Tauranga Hospital, Tauranga, New Zealand

**Keywords:** Human papillomavirus (HPV), Recurrent Respiratory Papillomatosis, RRP, aggressive disease, human immunodeficiency virus, HIV, dysplasia, age of onset

## Abstract

**Background:**

Recurrent respiratory papillomatosis can present with a highly variable clinical course. The disease can cause serious morbidity and can be fatal because of airway obstruction. We examined whether the age of onset, gender, human immunodeficiency virus (HIV) infection and dysplasia on analysis of histological specimens were predictive of an aggressive disease course.

**Objectives:**

To conduct an audit of all patients presenting with Recurrent Respiratory Papillomatosis at our institution and to determine if an earlier age of onset, gender, HIV and dysplasia are predictive factors for an aggressive disease course.

**Methods:**

A total of 202 clinical records and histological reports were reviewed at a quaternary-level hospital in Durban, South Africa. The disease was defined as juvenile onset (< 18 years) or adult onset (≥ 18 years). Aggressive disease was defined as a disease requiring 10 or more surgical debulkings in total and or extralaryngeal papilloma.

**Results:**

A total of 184 patients were of juvenile onset and 18 were of adult onset. In the juvenile onset group, a total of 97 patients (52.8%) had aggressive disease. In the juvenile onset group, a later age of onset was associated with less aggressive disease (odds ratio [OR] = 0.77, *p* < 0.05). There were 20 (10.9%) HIV-positive patients. HIV infection was a predictor of aggressive disease (OR = 3, *p* < 0.029). Analysis of histological reports revealed that 39 (21.2%) of patients had dysplasia. Dysplasia was a predictor of aggressive disease (OR = 9.96, *p* < 0.05%). In the adult onset group, only two patients (11.1%) had aggressive disease.

**Conclusion:**

An earlier age of onset, HIV infection and dysplasia were predictors of aggressive disease in the juvenile onset group.

## Introduction

Recurrent respiratory papillomatosis (RRP) remains a poorly understood disease that can be difficult to manage. Both juvenile onset RRP (JORRP) and adult onset RRP (AORRP) forms exist. The clinical course is highly variable with a spectrum from indolent to aggressive disease. It is the most common benign neoplasm affecting the larynx of children. Despite its benign nature, it can have potentially fatal consequences because of airway compromise. It is a chronic condition caused by the human papillomavirus (HPV).^1,2^

Early diagnosis, early surgical intervention and close follow-up are essential in managing the condition. Recurrent respiratory papillomatosis can have devastating consequences as it can be associated with significant morbidity with the potential for mortality as a result of airway obstruction. The disease imposes a significant burden on the patient, their family and community and adversely affects the patient’s quality of life. In this retrospective chart review, we examine whether an earlier age of onset, gender, human immunodeficiency virus (HIV) infection and dysplasia on analysis of histological specimens were predictive of an aggressive course of RRP. An earlier age of onset, gender and HIV infection are considered to be causative factors, while dysplasia is consistent as a marker of aggressive disease. These predictive factors are readily available to the clinician practicing in the developing world.

Three clinical patterns of RRP have been identified: JORRP, juvenile RRP persistent into adulthood and AORRP.^1,2,3^

The overall incidence of RRP has been determined in developed countries, and ranges from 0.6 per 100 000 to 2.13 per 100 000. The incidence in the United States is estimated at 4.3 per 100 000 children and 1.8 per 100 000 adults. The incidence in Denmark is estimated to be between 3.62 per 100 000 children and 3.94 per 100 000 adults.^1,2^ The incidence and prevalence of JORRP in the Free State province of South Africa was 1.34 per 100 000 population per year and 3.88 per 100 000 population, respectively. In AORRP, it was 0.18 per 100 000 population per year and 0.38 per 100 000 population, respectively.^4^ The incidence in sub-Saharan Africa is yet to be determined. The disease is more common in lower socio-economic settings and may present with more advanced disease in developing countries because of poor access to health care and delay in diagnosis. The known risk factors for acquiring respiratory papillomatosis as summarised in the 2017 article by Paolo Campisis include maternal age <20, the presence of genital warts during pregnancy, being first born, having a vaginal delivery of greater than >10 hours, as well as a specific subset of human leukocyte antigen (HLA) alleles.^5^ The emotional, social and economic burden of this disease is high, especially when repeated surgical debulkings are required.^6^ The estimated economic cost is $150 million annually in the United States.^7^ In the UK, the estimated total cost was $16.5 million over a 10-year period (1999–2009).^8^

The papillomaviruses are members of the papillomaviridae family. Over 150 HPV subtypes have been identified.^2^ The double-stranded circular deoxyribonucleic acid (DNA) molecule is an epitheliotropic virus that affects epithelial cells.^1^ Human papillomavirus-6 and HPV-11 are the subtypes that are most commonly associated with RRP. Human papillomavirus 11 appears to be at higher risk of obstructive airway disease. Human papillomavirus 16 and 18 are associated with malignancies of the genital and upper aerodigestive tract.^2,9^ However, some studies have shown no relationship between HPV type and aggressive disease.^1,2^

An association between HPV infection of the mother’s genital tract and RRP is well established. Human papillomavirus has been estimated to be present in 25% of all women of child bearing age worldwide. Human papillomavirus 6 and HPV 11 are the most commonly identified in cervical condyloma.^2,10^ Vertical transmission during delivery through an infected birth canal is thought to be the major mode of transmitting the infection in children, whereas *in utero* and transplacental transmission is thought to play a minor role. Seven out of 1000 children born to mothers with vaginal condylomata develop RRP.^2,3,10^

Maternal condylomatas are seen in more than 50% of mothers who give birth to children with RRP. Caesarean delivery of children seems to be preventative. Patients with childhood onset of RRP are more likely to be first-born and vaginally delivered in comparison to control patients of a similar age. Primigravid mothers are more likely to have a prolonged second stage of labour and the prolonged exposure leads to a higher risk of infection in the first-born child.^2,3,10^ Furthermore, newly acquired genital HPV lesions are more likely to shed HPV virus than long-standing lesions. This would explain the higher incidence of papilloma disease amongst offspring of younger mothers.^1,10,11^

While the presence of HPV is necessary for the development of RRP in observed cases, there does also appear to be a component of host susceptibility in whether or not an individual develops RRP, with the epidermodysplasia verruciformis 1(EVER1) as a postulated gene.^12^

The risk factors for AORRP are different from that of JORRP as the mode of transmission in these two disorders is different. Adult onset RRP is associated with an increased number of lifetime sexual partners and an increased frequency of oral sex.^13,14^

Respiratory papillomas predominantly affect the larynx. Other sites include the oral cavity, oropharynx, nasal cavities, nasopharynx, oesophagus, trachea and pulmonary spread. The average time between onset of symptoms and diagnosis is 1 year as reported in most paediatric series.^1,2^ The early stages of the disease present with progressive dysphonia (hoarse voice), and more advanced disease may present with stridor because of airway obstruction. Less common presenting symptoms include a chronic cough, recurrent pneumonia, failure to thrive in children, dyspnoea, dysphagia and acute respiratory distress.^1^

The clinical presentation and course of the disease is highly variable and unpredictable. The mean age of diagnosis is between the age of 3.6–6 years in the paediatric population and 20–30 years in the adult population. The mean age of diagnosis is thought to be higher in developing countries because of later presentation as a result of poorer access to health care.^1,2^ The gender distribution is equal in the paediatric population with a higher male preponderance in the adult population. A total of 5% – 48% of paediatric patients develop distal spread. The disease is characterised by repeated recurrences; however, juvenile onset patients may go into remission during puberty.^1,2,7,15,36^

There is no curative treatment. Management is with repeated microlaryngoscopic examinations with surgical removal of the papillomata until the disease goes into remission. The goal of surgical debulking is to excise the papillomatous lesions and create an adequate airway while maintaining the integrity of the underlying laryngeal tissue, especially the vocal cords. Methods for removal include cold steel technique using cupped forceps, laser excision (CO_2_ laser, KTP laser, 585 nm flash dye or argon laser), microdebrider and coblation.^1,2^ An advantage of laser excision is that the method allows for vaporisation of papillomata with minimal bleeding. A disadvantage is the risk of airway fires and scarring of the underlying tissues.^16^ The cold steel technique using cupped forceps is advantageous over laser surgery as it is associated with less scarring of the underlying laryngeal tissue. The endoscopic microdebrider technique has been shown to have improved outcomes over the CO_2_ laser with shorter operating times, less mucosal injury, improved voice outcomes and a cost benefit over CO_2_ laser.^16,17,18,19^ Coblation (radiofrequency ablation) is a promising surgical technique. The main advantages include limited damage to the underlying tissue and a bloodless field. In a retrospective chart series, use of the coblator resulted in longer periods between interventions as compared to CO_2_ laser.^20,21,39^ Despite removal of papillomata, the latent HPV viral DNA remains in the laryngeal tissue. Long-term complications include anterior laryngeal synechiae, glottic stenosis, granulation formation and poor voice quality.^1,2,22,23,24^

A tracheostomy is required when there is airway obstruction because of extensive papillomata, glottic and/or supraglottic stenosis because of repeated surgical debulkings with resultant scarring and fibrosis of the underlying laryngeal tissue. The need for tracheostomy is higher in developing countries and in the paediatric population as a result of poorer access to health care.^2^ Patients tend to present with more advanced disease and airway obstruction. Long-term tracheostomy is associated with an increased risk of distal spread of the disease. Early decannulation is therefore recommended.^2,25^

Adjuvant treatment is indicated in patients who require four or more surgical debulkings per annum, rapid recurrences of papilloma with short disease-free intervals and or distal spread of papilloma beyond the subglottis.^1,2^ Adjuvant therapy is required in 12.6% and 47.6% of patients.^16^ Adjuvant therapy includes interferon alpha, indole 3 carbinol, mumps vaccine, MMR vaccine, antivirals (acyclovir, cidofovir, ribavirin), celebrex and bevacizumab (Avastin). Interferon alpha was the first adjuvant therapy to be offered. The mechanism of action involves immune modulation and inhibition of viral protein synthesis. Interferon alpha is currently less commonly used because of multiple side effects.^1,2^ Intralesional injection of cidofovir is the most commonly used adjuvant therapy. The drug is a cytosine analogue and a potent inhibitor of viral replication. Nephrotoxicity is the most common adverse effect. Animal studies have shown a high level of carcinogenicity and reports of dysplasia have been reported in patients with RRP on cidofovir.^1,16,38^ Avastin is an angiogenesis inhibitor that reduces the growth of papilloma. Avastin used together with KTP laser is advocated because of their complimentary actions. Clinical success and fewer complications have been reported with this combination therapy.^16^

Human papillomavirus vaccines are designed to elicit an immune response against HPV gene products. Human papillomavirus-like particles (HPV virus-like particles [VLP]) can be generated by the synthesis and self-assembly *in vitro* of the major virus capsid protein L1.^26^ The bivalent vaccine is designed to protect against subtypes 16 and 18. The quadrivalent vaccine protects against subtypes 6, 11, 16 and 18. The US Food and Drug Administration (FDA) approved a nine-valent version of the HPV vaccine in 2014 covering subtypes 6, 11, 16, 18, 31, 33, 45, 52 and 58.^2,27,28^

The vaccination schedule was revised in 2015, and the World Health Organization recommends administering routine vaccination with one of the three versions of the vaccine to women aged 11–12 or up to age 26 if not previously vaccinated and to men aged 13–26 if not previously vaccinated.^29^ Human papillomavirus vaccines are most effective when administered before the onset of sexual activity. All three vaccines have shown efficacy of over 90% against persistent infection in phase III trials.^30^ In addition, both vaccines have shown evidence of cross protection against other HPV subtypes. Levels remain high for up to 5 years. Human papillomavirus vaccines can reduce the incidence of cervicovaginal disease and decrease the incidence of RRP. Given 10 years of availability and documented effectiveness of HPV vaccines, there remain wide global disparities in access to this intervention.^28^ In South Africa, the extended programme on immunisation (EPI) provides free vaccination of children. The Medicines Control Council (MCC) approved two HPV vaccines in 2008, and the bivalent vaccine, Cervarix (GSK Aspen), contains viral like particles against HPV 6 and 11. Gardasil (Merck Manual [MSD]) contains VLP of HPV types 6, 11, 16 and 18. In April 2014, the South African National Department of Health implemented a school-based HPV vaccination programme for all girls 9 years or older in grade 4 in public schools, targeting almost half a million girls. Preliminary data show excellent coverage of the targeted population with 91% and 93% vaccinated in the first round and second round, respectively.^4,31^

Dysplasia occurs in 20% of patients. Laryngeal dysplasia is a premalignant condition, with a wide variability of malignant transformation reported in the literature. Malignant transformation occurs in rare cases and is mostly associated with HPV 11. The overall malignant transformation rate is 14% and the mean time to malignant transformation is 5.8 years. The malignant transformation rate is higher with increased dysplasia grade. Severe dysplasia shows a 30% transformation rate and mild or moderate grade shows 11%. Treatment modality does not show significant effects.^32^ To the authors’ knowledge, the association of dysplasia as a prognostic factor for aggressive RRP is not well documented.

The identification of prognostic factors can assist in identifying patients who are at risk of developing a more aggressive clinical course. Identification of patients who are at risk allows for close surveillance, early intervention and prevention of serious morbidity and mortality. Aggressive disease may be defined as extralaryngeal spread and/or >10 debulkings in total.^1,2,3^ Some predisposing factors that are associated with an aggressive versus an indolent clinical course have been identified, but RRP remains a rare and incompletely described disease.^5^

An earlier age of onset, juvenile onset versus adult onset and genotype are all postulated as factors associated with an aggressive disease course. Several studies have found HPV 11 to be associated with more aggressive disease, whereas other studies have found no correlation between genotype and disease severity.^3,5,11^

The aim of this study is to describe the clinical course of RRP in paediatric and adult patients presenting to a quaternary-level hospital in the KwaZulu-Natal (KZN) province of South Africa and to determine whether the age of onset, HIV infection, gender and dysplasia on histological specimens are prognostic factors for developing an aggressive clinical course and complications. The above prognostic factors are readily accessible to the clinicians practising in the developing world.

## Materials and methods

### Study population and data collection

The study was carried out at Inkosi Albert Luthuli Central Hospital (IALCH) in the KZN province of South Africa. Inclusion criteria comprised all patients presenting to IALCH with histology confirmed RRP. The study period was from January 2005 to December 2014. Patients were identified through the hospital’s electronic medical records database (Meditec and Sorian Information Systems). The data that were extracted included the age of onset, gender, HIV infection, method of surgical debulking, tracheostomy, status at the end of the study period (active or remission or lost to follow-up or demised) and extralaryngeal papilloma. Aggressive disease was defined as the patients requiring a total of 10 or more surgical debulkings and/or extralaryngeal papilloma.

The data were then analysed to determine whether each of the following variables – age of onset, gender, HIV infection and or dysplasia – was associated with an aggressive disease course.

## Statistical analysis

Research data were primarily analysed within a quantitative framework using univariate and multivariate analyses. Data were analysed using Statistical Packages for the Social Sciences (SPSS) version 25. A *p*-value of < 0.05 was considered as statistically significant. Descriptive statistical analysis of the data (means, standard deviations, ranges, frequencies and percentages) was initially conducted prior to conducting inferential statistics. Logistic regression was used to assess the factors influencing the aggressiveness of RRP.

## Results

A total of 202 clinical records and histological reports were reviewed, of which 184 patients were of juvenile onset and 18 were of adult onset (Table 1). The majority of patients (195; 96.5%) were surgically debulked using the cold steel technique. Only the juvenile onset patients were debulked using alternative methods, of which only four (2%) patients were debulked using CO_2_ laser and three (1.5%) patients were debulked using the microdebrider technique. No adjuvant therapy was used. A tracheostomy was performed in 11 patients (5.4%). Indications for tracheostomy included supraglottic and glottic stenosis. One patient required an emergency tracheostomy because of airway obstruction. No tracheostomies were performed in the adult onset group.

**TABLE 1:** Comparison between juvenile onset and adult onset groups.

Variable	Number of patients	Aggressive disease		Mean age of onset (years)	Gender				HIV-positive		Dysplasia and dysplasia category							
		*n*	%		Men		Woman		*n*	%	Total		Mild		Moderate		Severe	
				*n*	%	*n*	%	*n*			%	*n*	%	*n*	%	*n*	%
JORRP	184	97	52.8	7.65	113	61.4	71	38.6	20	10.9	39	21.2	11	6	23	12.5	5	2.7
AORRP	18	2	11.1	30.6	7	38.9	11	61.1	8	44.4	2	11.1	-	-	Dysplasia	-	-	-

JORRP, juvenile onset recurrent respiratory papillomatosis; AORRP, adult onset recurrent respiratory papillomatosis; HIV, human immunodeficiency virus.

In the juvenile onset group, a total of 97 (52.8%) had aggressive disease. Sixty patients (32.6%) had extralaryngeal papilloma. Eighty-eight (43.6%) had less than 10 surgical debulkings. At the end of the study period, 73 (39.7%) patients were active, 97 (52.7%) had gone into remission, 13 (7.1%) were lost to follow-up and one (0.5%) had demised. In the adult onset group, only two patients (11.1%) had aggressive disease. Sixteen (88.9%) had gone into remission, and two (11.1%) patients were active at the end of the study period. No adult patients had demised during the study period (Table 1).

The mean number of surgical debulkings was 11.79 (standard deviation [s.d.] 14.20). The mean number of surgical debulkings in the juvenile onset group was 12.6 (s.d. 14.60). The mean number of surgical debulkings in the adult onset group was 3.4 (s.d. 3.4). The mean age of onset was 5.2 (s.d. 3.7) years in the juvenile onset group and 33 (s.d. 12) years in the adult onset group. In the juvenile onset group, a later age of onset was associated with less aggressive disease (odds ratio [OR] = 0.77, *p* < 0.05). The age of onset was not a predictor of aggressive disease in the adult onset group (OR = 83.34, *p* > 0.99).

In the juvenile onset group, there were 113 (61.4%) men and 71 (38.6%) women. Gender was not a predictor of aggressive disease (OR = 0.76, *p* = 0.45). In the adult onset group, there were 7 (38.9%) men and 11 (61.1%) women. Gender was also not a predictor of aggressive disease in the adult onset group (OR = 0.6, *p* > 0.99) (Tables 1 and 2).

**TABLE 2:** Gender, human immunodeficiency virus, dysplasia and age of onset as predictors of aggressive disease in the juvenile onset group.

Variable	Predictor of aggressive disease	Odds ratio	95% Confidence interval	*p*
Gender	No	0.76	0.41–1.4	0.449
HIV	Yes	3.0	1.1–7.8	0.029
Dysplasia	Yes	7.0	2.817	< 0.05
Age of onset	Yes	0.77	0.69–0.86	< 0.05

HIV, human immunodeficiency virus.

In the juvenile onset group, there were 20 (10.9%) HIV-positive patients and 164 (89.1%) HIV-negative patients, and HIV infection was a significant predictor of aggressive disease (OR = 3.0, *p* < 0.029). In the adult onset group, there were eight (44.4%) HIV-positive patients and 10 (55.6%) HIV-negative patients. Human immunodeficiency virus was not a significant predictor of aggressive disease in the adult onset group (OR 1.29, *p* > 0.71) (Tables 1 and 2).

**FIGURE 1:**
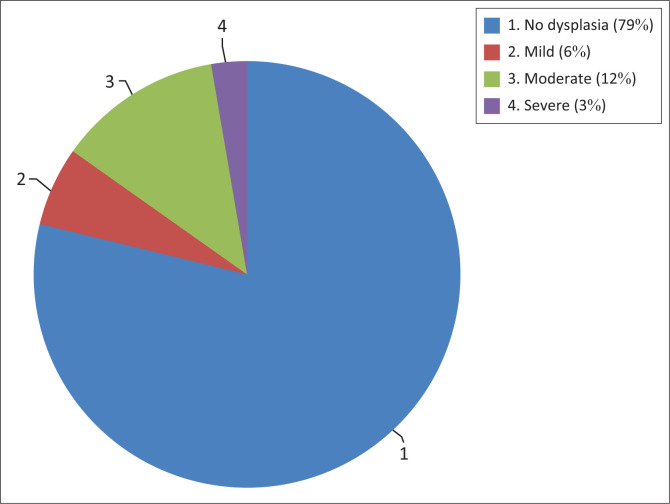
Dysplasia categorisation in the juvenile onset group.

Analysis of histological reports revealed that 39 (21.2%) of patients in the juvenile onset group had dysplasia. Eleven (6%) had mild dysplasia, 23 (12.5%) had moderate dysplasia and five (2.7%) had severe dysplasia (Figure 1). Dysplasia was a predictor of aggressive disease in the juvenile onset group (OR = 6.96, *p* < 0.05%). In the adult onset group, only two (11.1%) had dysplasia and this was moderate dysplasia. Dysplasia was not a predictor of aggressive disease in the adult onset group (OR 15, *p* = 0.216) (Tables 1 and 2).

## Discussion

To our knowledge, this is the first study in the KZN province of South Africa to determine the risk factors associated with an aggressive disease course of RRP. The cohort size of 202 cases over a 10-year period suggests an impressive burden of disease in the province and is one of the largest reported in the literature. By comparison, the entire US National Registry for JORRP contained only 603 patients as of 2003 and the Canadian national database identified 243 cases of JORRP nationally. The disease predominantly affects the larynx, and as a result, patients are at risk of developing serious morbidity because of airway obstruction. Identification of risk factors for aggressive disease is important to determine the frequency of follow-up visits and early surgical intervention to prevent serious complications.

We defined aggressive disease as disease requiring 10 or more surgical debulkings and or extralaryngeal spread of papilloma.^2^ The age of onset was defined as the age of first presentation with surgical debulking and histological confirmation. The mean age of onset was 5.2 years in the paediatric population and 30.6 years in the adult population. An older age of diagnosis in the paediatric population is in line with studies from developing countries.^25^ In our paediatric cohort of patients, we found that a later age of onset was associated with less aggressive disease (OR = 0.77, *p* < 0.05).

A smaller glottic diameter in younger patients can result in the need for more frequent debulkings, qualifying for a diagnosis of aggressive disease. Other studies have speculated that the developing immune system may permit older children to have slower papilloma growth. Patients who present at an earlier age of onset are at greater risk of developing aggressive disease and need to be closely monitored with regular follow-up visits and early surgical intervention.^12,33^

Analysis of histological reports found that dysplasia occurred in 21.2% of patients in the paediatric cohort, which is in keeping with international literature. Dysplasia on histological analysis was a predictor of aggressive disease in the paediatric cohort. Furthermore, given the risk of malignant transformation, patients with dysplasia need to be followed up for a period of at least 5 years after going into remission. Even after discharge, patients should be carefully counselled on early warning signs of malignancy.^32^

In the juvenile onset group, there were 20 (10.9%) HIV-positive patients. The cluster of differentiation 4 (CD4) count varied from 224 to 1215. A depressed immune system is a known risk factor for developing RRP. HIV infection proved to be a marginally significant predictor for aggressive disease in our paediatric cohort of patients. Hence, patients who are HIV-positive require close surveillance and early surgical debulking.^34^

In the juvenile onset group, there were 113 (61.4%) men and 71(38.6%) women. Gender was not a predictor of aggressive disease in our cohort of patients, which is in line with international literature. Most studies have found an equal gender distribution.^1,2,3^

Our adult onset group was very small, with only 18 patients. Only two (11.1%) had aggressive disease. The age of onset, gender, HIV infection or dysplasia were not predictors of aggressive disease in the adult onset group, but this was probably limited because of the small sample size.

A limitation of this study is that we did not have access to HPV genotyping because of cost factors. However, some studies have shown no relationship between HPV types and disease aggressiveness.^37^ Furthermore, an aggressive disease course is associated with HPV type and age at onset. Once the age of the child is controlled for, then the relationship between HPV type and clinical course becomes of borderline significance.^35^

None of the patients in our cohort were vaccinated at the time of the study. Although HPV vaccination programmes have been rolled out in certain public schools, large sectors of the population remain unvaccinated worldwide. In a resource-limited, developing world setting, it is useful to identify clinical and histological parameters that predict aggressive disease and prevent fatal consequences.

## Conclusion

Recurrent respiratory papillomatosis presents with a highly variable clinical course in both juvenile and adult patients, with a spectrum from indolent to aggressive disease. All patients need to be monitored with regular follow-up visits and early surgical debulking when indicated. Patients with aggressive disease require close surveillance in order to prevent the devastating consequences of airway obstruction. Laryngeal dysplasia is a premalignant condition, with a wide variability of malignant transformation reported in the literature; however, to our knowledge, the association between dysplasia and severe respiratory papilomatosis is poorly documented. In our paediatric cohort of patients, an earlier age of onset, dysplasia on histological analysis and HIV infection were all predictive factors of aggressive disease.

## References

[1] Derkay CS, Wiatrak B. Recurrent respiratory papillomatosis, a review. Laryngoscope. 2008;118:1236–1247. 10.1097/MLG.0b013e31816a7135 18496162

[2] Seedat RY, Combrinck CE, Burt FJ. HPV associated with recurrent respiratory papillomatosis. Future Virol. 2013;8(5):477–492. 10.2217/fvl.13.31

[3] Omland T, Akre H, Lie KA, Jebsen P, Sandvik L, Brondbo K. Risk factors for aggressive recurrent respiratory papillomatosis in adults and juveniles. PLoS One. 2014;9(11). 10.1371/journal.pone.0113584 PMC4242649 25419846

[4] Moodley I, Tathiah N, Mubaiwa V, Denny I. High uptake of Gardasil vaccine among 9 to 12-year-old schoolgirls participating in an HPV vaccination demonstration project in KwaZulu-Natal, South Africa. S Afr Med J. 2013;103(5):318–321. 23971122 10.7196/samj.6414

[5] Campisi P. The epidemiology of recurrent respiratory papillomatosis. Recurr Respirat Papillomatosis. 2017;10:19–31.

[6] Lindman JP, Lewis LS, Accortt N, Wiatrak BJ. Use of the paediatric quality of life inventory to assess the health-related quality of life in children with recurrent respiratory papillomatosis. Ann Otol Rhinol Laryngol. 2005;114:499–503. 10.1177/000348940511400701 16134343

[7] Derkay C. Task force on recurrent respiratory papillomatosis. A preliminary report. Arch Otolaryngol Head and Neck Surg. 1995;121:1386–1391. 7488368 10.1001/archotol.1995.01890120044008

[8] Hughes OR. Toward prevention or cure for recurrent respiratory papillomatosis. Laryngoscope. 2010;122(Suppl. 4):S63–S64. 10.1002/lary.23805 23254606

[9] Penaloza-Plascencia M, Montoya-Fuentes H, Flores-Martinez SE, Fierro-Velasco FJ, Penaloza Gonazalez JM, Sanchez-Corona J. Molecular identification of 7 human papillomavirus types in recurrent respiratory papillomatosis. Arch Otolaryngol Head Neck Surg. 2007;126(9):1119–1123. 10.1001/archotol.126.9.1119 10979126

[10] Shah KV, Stern WF, Shah FK, Bishai D, Kashima HK. Risk factors for juvenile onset recurrent respiratory papillomatosis. Pediatr Infect Dis J. 1998;17:372–376. 9613648 10.1097/00006454-199805000-00005

[11] Kashima HK, Shah F, Lyles A. A comparison of risk factors in juvenile onset and adult onset recurrent respiratory papillomatosis. Laryngoscope. 1992;102:9–13. 10.1288/00005537-199201000-00002 1309932

[12] Seedat RY, Schall R. Age of diagnosis, incidence and prevalence of recurrent respiratory papillomatosis-A South African perspective. 2018. Clin Otolaryngolo. 2018;43(2):533–537. 10.1111/coa.13016 29054106

[13] Gabbott M, Cossart YE, Kan A, Konopka M, Chan R, Rose BR. Human papillomavirus and host variables as predictors of clinical course in patients with juvenile onset recurrent respiratory papillomatosis. J Clin Microbiol. 1997;35(12):3098–3108. 9399501 10.1128/jcm.35.12.3098-3103.1997 PMC230129

[14] Ruiz R, Achlatis S, Verma A, et al. Risk factors for adult onset recurrent respiratory papillomatosis. Laryngoscope. 2014;124(10):2338–2344. 10.1002/lary.24730 24764146

[15] Armstrong LR, Derkay CS, Reeves WC. Initial results from the national registry for Juvenile Onset Recurrent Respiratory Papillomatosis. Arch Otolaryngol Head Neck Surg. 1999;125:743–748. 10406310 10.1001/archotol.125.7.743

[16] Avelino MAG, Zaiden TCDT, Gomez RO. Surgical treatment and adjuvant therapies of recurrent respiratory papillomatosis. Braz J Otorhinolaryngol. 2013;79(5):636–642. 10.5935/1808-8694.20130114 24141682 PMC9442437

[17] Pasquale K, Wiatrak B, Woolley A, Lewis L. Microdebrider versus CO_2_ laser removal of recurrent respiratory papillomas: A perspective analysis. Laryngoscope 2003;113:139–143. 10.1097/00005537-200301000-00026 12514398

[18] Patel N, Rowe M, Tunkel D. Treatment of recurrent respiratory papillomatosis in children with the microdebrider. Ann Otol Rhinol Laryngol. 2003;112:7–10. 10.1177/000348940311200102 12537050

[19] EL-Bitar MA, Zalzal GH. Powered instrumentation in the treatment of recurrent respiratory papillomatosis: An alternative to the CO_2_ laser. Arch Otolaryngol Head Neck Surg. 2002;128:425–428. 11926919 10.1001/archotol.128.4.425

[20] Rachmanidou A, Modayil PC. Coblation resection of paediatric laryngeal papilloma. J Laryngol Otol. 2011;125(8):873–876. 10.1017/S0022215111001253 21669019

[21] Carney AS, Evans AS, Mirza S, Psaltis A. Radiofrequency coblation for treatment of advanced laryngotracheal recurrent respiratory papillomatosis. J Laryngol Otol. 2009;125(5):510–514. 10.1017/S0022215109992398 20003595

[22] Dean C, Sataloff RT, Hawkshaw M. Recurrent vocal fold papilloma: resection using cold instruments. Ear Nose Throat J. 1998;77:882–884. 9846463

[23] Uloza V. The course of laryngeal papillomatosis treated by endolaryngeal microsurgery. Eur Arch Otorhinolaryngol. 2000;257:498–501. 11131377 10.1007/s004050000273

[24] Zeitels SM, Sataloff RT. Phonomicrosurgical resection of glottal papillomatosis. J Voice. 1999;13:123–127. 10.1016/S0892-1997(99)80066-6 10223680

[25] Orji FT, Okorafor IA, Akpeh JO. Experience with recurrent respiratory papillomatosis in a developing country: Impact of tracheostomy. World J Surg. 2013;37(2):339–343. 10.1007/s00268-012-1839-y 23135424

[26] Stanley M. Prophylactic HPV vaccines. Drugs today (Barc). 2007;43(10):737–744. 10.1358/dot.2007.43.10.1136900 17987226

[27] Cutts FT, Franceschi S, Goldie S et al. Human papillomavirus and HPV vaccines: A review. Bull. World Health Organ. 2007;85(9):719–726. 10.2471/blt.06.038414 18026629 PMC2636411

[28] Bloem P, Ogbuanu I. Vaccination to prevent human papillomavirus infections: From promise to practice. PLOS Med. 2017;14(6): e1002325. 10.1371/journal.pmed.1002325 28654640 PMC5486966

[29] Petrosky E, Bocchini JA, Hariri S, et al. Use of 9 valent Human papillomavirus (HPV) Vaccine: updated HPV vaccination recommendations of the advisory committee on immunization practices. MMWR Morb Mortal Wkly Rep. 2015;64(11):300–304. 25811679 PMC4584883

[30] Tabrizi SN, Garland SM. A prospective study on the incidence of juvenile onset recurrent respiratory papillomatosis after implementation of a national HPV vaccination program. J Infect Dis. 2018;218(1):95–108. 10.1093/infdis/jix498 29136168

[31] Richter K. Implementation of HPV vaccination in South Africa. [serial online]. 2015 [cited 2019 Oct 31]; 1–4. Available from: PHASA. https://www.phasa.org.za/wp-content/uploads/2015/02/Richter_Implementation-of-HPV_article-4.pdf.

[32] Weller MD, Nankivell PC, McConkey C, Paleri V, Mehanna HM. The risk and interval to malignancy of patients with laryngeal dysplasia; a systematic review of case series and meta-analysis. Clin Otolaryngol. 2010;35:364–372. 10.1111/j.1749-4486.2010.02181.x 21108746

[33] Gale N, Poljak M, Kambic V, Ferluga D, Fischinger J. Laryngeal papillomatosis: molecular, histopathological and clinical evaluation. Virchows Arch. 1994;425(3):291–275. 7812515 10.1007/BF00196152

[34] Sajan JA, Kerschner JE, Merati AL, Osipov V, Szabo S, Blumin JH. Prevalence of dysplasia in juvenile onset recurrent respiratory papillomatosis. Arch otolaryngol Head Neck Surg. 2010;136(1):7–11. 10.1001/archoto.2009.179 20083770

[35] Buchinsky FJ, Donfack J, Derkay CS, et al. Age of child, more than HPV type, is associated with clinical course in recurrent respiratory papillomatosis. PLoS One. 2008;3(5):1–8. 10.1371/journal.pone.0002263 PMC2386234 18509465

[36] Lindeberg H, Elbrond O. Laryngeal papillomas: clinical aspects in a series of 231 patients. Clin Otolaryngol. 1989;14(4):333–342. 2805372 10.1111/j.1365-2273.1989.tb00381.x

[37] Abramson AL, Steinberg BM, Winkler B. Laryngeal papillomatosis: clinical, histopathological and molecular studies. Laryngoscope. 1987;97(6):678–685. 3035299 10.1288/00005537-198706000-00005

[38] Snoeck R, Wellens W, Desloovere C. Treatment of severe laryngeal papillomatosis with intralesional injections of cidofovir. [(S)-1-(3-Hydroxy-2-phosphonylmethoxypropyl)cytosine]. J Med Virol. 1998;54:219–225. 9515772 10.1002/(sici)1096-9071(199803)54:3<219::aid-jmv13>3.0.co;2-c

[39] Rachmanidou A, Modayil PC. Coblation resection of paediatric laryngeal papilloma. J Laryngol Otolo. 2011;125(8):873–876. 10.1017/S0022215111001253 21669019

